# Mechanism of Resistance to Dietary Cholesterol

**DOI:** 10.1155/2011/101242

**Published:** 2011-10-05

**Authors:** Lindsey R. Boone, Patricia A. Brooks, Melissa I. Niesen, Gene C. Ness

**Affiliations:** ^1^Department of Pathology, McGowan Institute for Regenerative Medicine, University of Pittsburgh, Pittsburgh, PA 15219, USA; ^2^Department of Molecular Medicine, College of Medicine, University of South Florida, Tampa, FL 33612, USA

## Abstract

*Background*. Alterations in expression of hepatic genes that could contribute to resistance to dietary cholesterol were investigated in Sprague-Dawley rats, which are known to be resistant to the serum cholesterol raising action of dietary cholesterol. *Methods*. Microarray analysis was used to provide a comprehensive analysis of changes in hepatic gene expression in rats in response to dietary cholesterol. Changes were confirmed by RT-PCR analysis. Western blotting was employed to measure changes in hepatic cholesterol 7**α** hydroxylase protein. *Results*. Of the 28,000 genes examined using the Affymetrix rat microarray, relatively few were significantly altered. As expected, decreases were observed for several genes that encode enzymes of the cholesterol biosynthetic pathway. The largest decreases were seen for squalene epoxidase and lanosterol 14**α** demethylase (CYP 51A1). These changes were confirmed by quantitative RT-PCR. LDL receptor expression was not altered by dietary cholesterol. Critically, the expression of cholesterol 7**α** hydroxylase, which catalyzes the rate-limiting step in bile acid synthesis, was increased over 4-fold in livers of rats fed diets containing 1% cholesterol. In contrast, mice, which are not resistant to dietary cholesterol, exhibited lower hepatic cholesterol 7**α** hydroxylase (CYP7A1) protein levels, which were not increased in response to diets containing 2% cholesterol.

## 1. Introduction

There is considerable variation among animals and humans in terms of their responses to consumption of excess dietary cholesterol. Consumption of a high-cholesterol diet does not automatically result in elevated serum cholesterol levels due to the operation of adaptive responses. Rabbits, hamsters, and C57BL/6 mice are not resistant to dietary cholesterol and exhibit marked elevations in serum cholesterol levels when given diets supplemented with cholesterol [1–3]. On the other hand, rats such as Sprague-Dawley, Wistar-Furth, Spontaneously Hypertensive or Fischer 344 show very little if any increase in serum cholesterol levels when given a similar cholesterol challenge [2]. For most humans, consumption of increased amounts of dietary cholesterol produces only small increases in both LDL and HDL cholesterol with little effect on the ratio of LDL to HDL [4, 5]. In a 14-year study of over 80,000 female nurses, egg consumption was unrelated to the risk of coronary heart disease [4]. On balance, extensive epidemiologic studies show that dietary cholesterol is not a contributor to increased heart disease risk in humans [6]. Clearly, in many rat strains and most people, adaptive responses are operating that keep serum cholesterol levels within the normal range. 

Multiple possible mechanisms may be operating to provide a person or animal with resistance to the serum cholesterol-raising action of dietary cholesterol. These include decreasing the rate of cholesterol biosynthesis, decreasing the rate of cholesterol absorption, increasing the rate of cholesterol excretion, increasing the conversion of cholesterol to bile acids, and increasing the rate of removal of serum cholesterol via liver lipoprotein receptors [7, 8]. 

In order to obtain a comprehensive and unbiased analysis of adaptive responses in hepatic gene expression to dietary cholesterol, we carried out microarray analysis of changes in Sprague-Dawley rat liver gene expression elicited by a 1% cholesterol diet. These animals are known to have adaptive responses that render them resistant to dietary cholesterol. In order to induce atherosclerotic plaques in these animals, the rats must be rendered hypothyroid [9]. Thus, this animal is a reasonable model for human responses to dietary cholesterol. In a previous microarray study, C57BL/6 mice were used [10]. These mice become hypercholesterolemic and develop fatty lesions in their ascending aortas when given diets supplemented with cholesterol [11, 12]. 

Liver was selected for extensive study, because this tissue not only synthesizes cholesterol but also is also responsible for bile acid production and excretion of cholesterol and expresses the majority of the body's LDL receptors [13].

## 2. Methods

### 2.1. Animals

Male Sprague-Dawley rats, 150–200 g (Harlan, Madison, Wis, USA), were fed Harlan Teklad 22/5 rodent chow with or without 1% cholesterol for five days. The animals were kept in a reversed lighting cycle room (12 dark/12 hrs light) and sacrificed at 0900–1000 hrs, corresponding to the third to fourth hour of the dark period when cholesterol biosynthesis is maximal. Twelve-week old C57BL/6J mice were fed chow with or without 2% cholesterol for 5 days. Liver samples were obtained, and microsomes were prepared from both rats and mice [14]. All procedures were carried out according to the regulations and oversight of the University of South Florida Institutional Animal Care and Use Committee (IACUC), protocol 2953.

### 2.2. Cholesterol Analysis

Trunk blood was collected and allowed to clot. The samples were centrifuged at 5,000 xg for 5 min. The serum was removed with a Pasteur pipette. Total serum cholesterol levels were determined by a cholesterol oxidase method using Infinity Cholesterol Reagent (Sigma) [15]. Values are expressed as mg/dL of serum. Liver cholesterol levels were determined as previously described [16]. Briefly, weighed liver samples were saponified, extracted with petroleum ether and subjected to reverse-phase high-performance liquid chromatography on a Spheri-5, RP-18, 5 reverse-phase column (Altech Associates). Values are expressed as mg/g of liver.

### 2.3. RNA Isolation

A portion of about 200 mg was quickly excised from livers of the rats and immediately homogenized in 4 mL of Tri-Reagent from Molecular Research Center (Cincinnati, Ohio, USA) using a Polytron homogenizer at room temperature. The remainder of the isolation steps was carried out using volumes corresponding to 4x the manufacturer's recommendations. RNA concentrations were determined by diluting each sample 1 : 100 and measuring its absorbance at 260 nm.

### 2.4. Microarray Analysis 

Isolated RNA was further purified using the RNeasy kit from Qiagen. To demonstrate that the RNA was indeed free of RNase activity, samples were incubated in water at 42°C for 1 hr and then examined on 1% agarose gels. An identical pattern of bands in unheated and heated samples was obtained showing a lack of RNase activity. Microarray analysis was performed by the Moffitt Core Facility (Tampa, FL) using the Affymetrix GeneChip Instrument system and the protocol established by Affymetrix, Inc. Ten *μ*g of RNA each from the livers of 3 control and 3 cholesterol-fed rats was used in the analysis. The RNA was converted to double-stranded cDNA using an oligo(dT)24 primer containing a T7 RNA polymerase recognition sequence. The product was transcribed into biotin-labeled cRNA using T7 RNA polymerase. The biotinylated cRNA was hybridized to Affymetrix GeneChip Rat Genome 230 Plus 2.0 arrays, which detects about 28,000 genes. Multiple oligos were used for each gene with the data averaged. Scanned chip images were analyzed using GeneChip algorithms.

### 2.5. Real Time RT-PCR Analysis

To validate the microarray results, we assessed the expression of a subset of genes via real-time PCR essentially as described previously [14]. Total RNA was resuspended in diethyl pyrocarbonate-treated water. Twenty micrograms of RNA was then DNAse-treated using the TURBO DNA-Free Kit from Ambion. cDNA was prepared from 1 *μ*g of DNAse-treated RNA using the Reverse Transcription System from Promega. The final product was brought up to 100 *μ*L, and a total of 2 *μ*L of the reverse transcription reaction was then used for real-time PCR analysis. The primer sequences used are given in Table 1. PCR was carried out according to the protocol from ABI Power SYBR Green Master Mix, using a Chromo-4 DNA Engine (Bio-Rad) with minor modifications. The program used for amplification was (i) 95°C for 5 minutes, (ii) 95°C for 15 seconds, (iii) 61°C for 1 minute (collect data), and (iv) go to step (ii) 40 times, (v) 55°C + 0.5°C each 10 seconds, ×80 (melt curve). The results were quantified by the ΔΔC_t_ method using Microsoft Excel statistical programs and SigmaPlot 8.0. As a housekeeping gene, 18S ribosomal RNA was used. Values are expressed relative to this.

### 2.6. Western Blot Analysis

At time of sacrifice, a portion of liver was excised for protein analysis. Lysosome-free liver microsomes were prepared according to the procedure previously described [17]. Liver microsomes (50 *μ*g of protein per lane) from rats and mice were subjected to SDS-PAGE and western blotting. Membranes were incubated overnight with 1 : 2,000 dilution of cholesterol 7*α* hydroxylase primary antibody generated in rabbits and generously provided to us by Dr. Mats Rudling in 5% PBST nonfat dry milk. A 1 : 10,000 dilution of Sheep anti Rabbit IGG was as the secondary antibody. The West Pico Chemiluminesence kit was used for detection with exposure times ranging from 5 to 20 seconds. The blots were then stripped and reprobed with an antibody to *β*-actin. The resulted were expressed relative to the *β*-actin signal.

### 2.7. Statistics

For the microarray data, a 2-tailed distribution *t*-test, equal variance was used. The RT-PCR data are presented as means ± standard errors. *P* values using a 2-tailed distribution *t*-test are given.

## 3. Results

### 3.1. Microarray Analysis

In order to comprehensively identify the genes that exhibit significant alterations in rates of transcription in response to dietary cholesterol, microarray analysis was performed on hepatic RNA from Sprague-Dawley rats fed either normal rodent chow or chow supplemented with 1% cholesterol for 5 days. This dietary regimen does not raise serum cholesterol levels in these male Sprague Dawley rats [2, 18]. Liver cholesterol levels are increased about 2-fold (Table 2). This is similar to our previous findings with 2% cholesterol [16]. Thus, this treatment appears to constitute a useful model for studying adaptive responses.

Surprisingly, the rates of transcription of relatively few genes were altered more than 2-fold (Table 3). The largest increase was observed for insulin-like growth factor-binding protein 1. This increase of over 4-fold was confirmed by RT-PCR analysis (Table 5). A prominent increase of nearly 3-fold in hepatic cholesterol 7*α* hydroxylase expression was observed. This would provide for increased production of bile acids and more efficient elimination of biliary cholesterol, since this enzyme catalyzes the rate-limiting step of bile acid synthesis. ABCG5 and ABCG8 expression was actually decreased (Table 4). These ATP-binding cassette (ABC) transport proteins promote biliary secretion of neutral sterols [19]. Also, acetyl CoA acetyltransferase 2 (ACAT2), which esterifies excess cholesterol, was significantly decreased. Strikingly, there was no change in hepatic LDL receptor expression. 

Many of the genes exhibiting downregulated expression in response to dietary cholesterol (Table 3) catalyze reactions of the cholesterol biosynthetic pathway. Levels of mRNA for the enzyme that catalyzes the rate-limiting reaction, HMG-CoA reductase, were only decreased 2-fold. RT-PCR analysis (Table 4) showed only a 1.4-fold decrease HMG-CoA reductase, in agreement with previous results from Northern blotting analysis [20]. The largest decrease (7.9-fold) was observed for squalene epoxidase. This enzyme regulates lanosterol synthesis [21]. The next largest decrease among cholesterol biosynthetic enzymes was seen for lanosterol 14*α* demethylase. The expression of several cholesterol biosynthetic enzymes including Δ14 sterol reductase, sterol C4 methyl oxidase-like, 7-dehydrocholesterol reductase, HMG-CoA synthase 1, farnesyl diphosphate synthase, lanosterol synthase, acetoacetyl CoA synthase, and mevalonate kinase were decreased 2 to 4-fold (Table 4). Other cholesterol biosynthetic enzymes such as sterol-C5-desaturase, phosphomevalonate kinase, diphosphomevalonate decarboxylase, 24-dehydrocholesterol reductase and dehydrogenase/reductase (SDR family) member 7 were decreased 1.5- to 1.9-fold. Other genes of interest whose expression was decreased by dietary cholesterol were SREBP-2 (1.4-fold, *P* = 0.028) and PCSK9 (1.5-fold, *P* = 0.022).

### 3.2. RT-PCR Analysis

Quantitative RT-PCR was utilized to examine a subset of the altered genes that were identified via microarray analysis. The results are presented in Table 5 and correlate with those seen in the microarray analysis. For example, the expression of squalene epoxidase (Sqle) and lanosterol 14*α* demethylase (Cyp51A1) were decreased by 8.29 and 4.53-fold, respectively (Table 5) as compared with 7.9 and 3.5-fold in the microarray analysis (Table 4). The relative fold changes in ABCG5 and 8, HMG-CoA synthase, ACAT-2, lanosterol synthase, sterol C4 methyl oxidase-like, lipin 2, cholesterol 7*α* hydroxylase, and 7-dehydrocholesterol reductase also agree very closely, providing further verification of the data obtained from the microarray analysis.

### 3.3. Western Blotting Analysis

**Figure 1:**
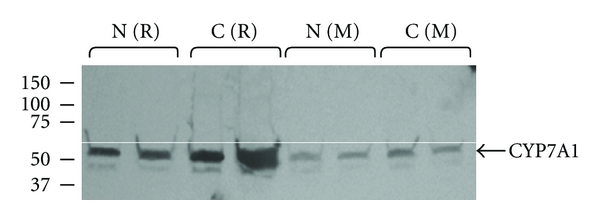
Comparison of the effects of dietary cholesterol on hepatic cholesterol 7*α* hydroxylase protein levels in rats and Mice. Rats (R) and mice (M) were fed normal (N) chow diets with or without 1% or 2% cholesterol (C) for 5 days. A representative Western blot of hepatic microsomal cholesterol 7*α* hydroxylase protein is presented.

Since the increase in rat liver cholesterol 7*α* hydroxylase mRNA caused by dietary cholesterol could be important for the elimination of cholesterol from the body, we wished to determine whether levels of this protein are actually increased. For comparison purposes, we examined the effect of dietary cholesterol on mouse liver cholesterol 7*α* hydroxylase protein. Cholesterol 7*α* hydroxylase protein levels were much higher in rat liver than in mouse liver (Figure 1). Supplementing the chow with 1% cholesterol increased hepatic cholesterol 7*α* hydroxylase protein levels in rats from 1.22 ± 0.78 to 6.52 ± 2.27 (4 chow-fed rats compared with 5 cholesterol-fed animals with a *P* = 0.011). In contrast, little effect on the levels in mouse liver was seen even when 2% cholesterol was added to the diet (Figure 1).

### 3.4. Time-Course Experiment

**Figure 2:**
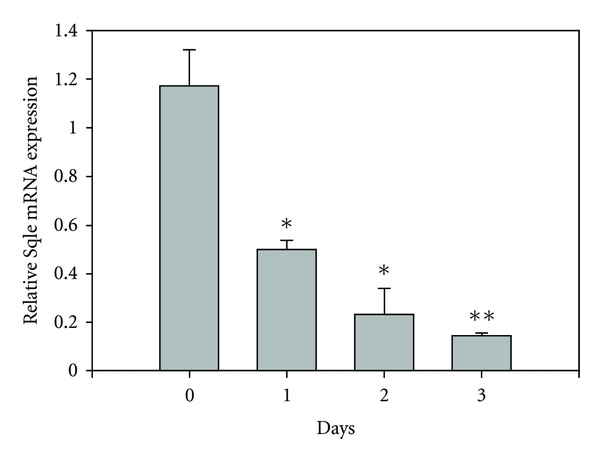
Effect of cholesterol feeding on hepatic squalene epoxidase mRNA levels. Rats were fed a diet containing 1% cholesterol for the indicated number of days. Hepatic mRNA levels were determined by RT-PCR. Values are given as means ± Standard Error for 3 to 6 rats per time point. *P < 0.05; **P < 0.01.

**Figure 3:**
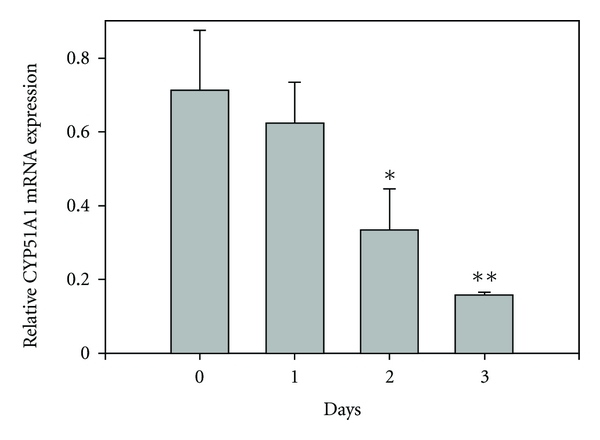
Effect of cholesterol feeding on hepatic lanosterol 14*α* demethylase mRNA levels. Rats were fed a diet containing 1% cholesterol for the indicated number of days. Hepatic mRNA levels were determined by RT-PCR. Values are given as means ± Standard Error for 3 to 6 rats per time point. *P < 0.05; **P < 0.01.

A time-course experiment was conducted to determine how rapidly dietary cholesterol reduces the expression of Sqle and Cyp51. RT-PCR analysis, as shown in Figures 2 and 3, demonstrates that the decrease in Sqle occurred more rapidly. Both Sqle and Cyp51 decreased progressively over a 3-day period. This agrees with the time course for the reduction in translation of HMG-CoA reductase mRNA [22].

## 4. Conclusion

Sprague Dawley rats from Harlan fed the grain-based Tekland 22/5 rodent chow diet resist a high cholesterol diet-induced increase in serum cholesterol as reported previously [2, 16]. They do show increases in liver cholesterol levels (Table 2). This indicates that dietary cholesterol is effectively taken up by the intestine, where Niemann-Pick C1-like 1 protein facilitates its uptake [23]. The resulting chylomicron remanents are taken up by the liver. With the increased cholesterol from the diet, the activity of the hepatic LDL receptor, as determined from its rate of turnover, is markedly decreased [18]. Thus, the cycling of this receptor, located in the cholesterol-rich caveolae portion of the plasma membrane [24], virtually stops [18]. The levels of hepatic LDL receptor protein and mRNA do not, however, change [18]. Since the liver must play a key role in adapting to and resisting the increase load of cholesterol, we conducted a microarray study to investigate changes in hepatic mRNA levels. 

Of all the hepatic genes examined, the most dramatic changes seen in response to dietary cholesterol were the decreases in Sqle and Cyp51. These changes seen in the microarray experiment were confirmed by RT-PCR. When lanosterol levels are decreased as occurs when Sqle expression is decreased, the different Kms of the three reactions catalyzed by Cyp51 come into play. The Km for the formation of 3*β*-hydroxylanosterol-8-en-32-al is 56 *μ*M while the Km for its destruction (conversion to 4 dimethylcholesta 8, 14 dien-3*β*-ol) is 368 *μ*M [25]. Although there would be sufficient lanosterol for the first reaction catalyzed by CYP51 (formation of 3*β*-hydroxylanosterol-8-en-32-al), because of the low Km, its subsequent conversion to 4 dimethylcholesta 8, 14 dien-3*β*-ol would be slow due to the high Km for this reaction. Thus, 3*β*-hydroxylanosterol-8-en-32-al would accumulate. This oxylanosterol is known to act to decrease the rate of translation of hepatic HMG-CoA reductase in CHO cells [26]. The structural analogue, 15*α*-fluoro-3*β*-hydroxylanost-7-en-32-aldehyde has also been reported to inhibit translation of HMG-CoA reductase mRNA in CHO cells [27]. In addition, it has been demonstrated that feeding the nonmetabolizable oxylanosterol analogue, 15 oxa-32-vinyllanost-8-ene-3*β*, 32 diol to rats results in a marked decrease in translation of hepatic HMG-CoA reductase mRNA which mimics the effect of dietary cholesterol [28]. Thus, dietary cholesterol-mediated decreases in Sqle and Cyp51 transcription lead to decreased translation of hepatic HMG-CoA reductase mRNA.

In this microarray study, a 2-fold decrease in HMG-CoA reductase expression was observed in response to dietary cholesterol. The SREs in the promoters of both Sqle and Cyp51 [29, 30] have been shown to be responsive to SREBP2 and would be expected to be downregulated by cholesterol feeding. However, the SRE sequence in the HMG-CoA reductase promoter does not agree well with the consensus SRE, which may explain the only modest decrease in HMG-CoA reductase transcription in response to dietary cholesterol. Previous Northern blotting analysis and nuclear run-on studies showed only slight decreases in mRNA levels and rates of transcription for hepatic HMG-CoA reductase [20, 31]. However, enzyme activity and protein levels drop to a couple percent of control values [20, 31]. This is due primarily to a marked decrease in the rate of translation of HMG-CoA reductase mRNA caused by feeding cholesterol [22]. The decrease in the rate of translation of HMG-CoA reductase mRNA is due to shift from association of the mRNA with polysomes to monosomes caused by dietary cholesterol [32]. The present observations are predicted by the proposed two-step model for feedback regulation of hepatic HMG-CoA reductase gene expression [33]. In this model, increased hepatic cholesterol leads to decreased formation of mature SREBP2 and decreased transcription of Sqle and Cyp51. This in turn results in accumulation of 3*β*-hydroxylanosterol-8-en-32-al, which acts to decrease translation of hepatic HMG-CoA reductase mRNA [20].

In addition to adapting to excess dietary cholesterol by decreasing the rate of hepatic cholesterol biosynthesis, adaptations in bile acid pathways also occurred. A 2.7-fold increase was observed in cholesterol 7*α* hydroxylase expression, which is the enzyme that catalyzes the rate-limiting step in bile acid synthesis (Table 3). We have previously observed by Northern blotting analysis that hepatic cholesterol 7*α* hydroxylase mRNA is markedly increased in rats [16]. Paradoxically, the microarray analysis did not show increases in expression of the ABCG5/G8 transporters. Instead, decreases in the expression of both ABCG5 and ABCG8 were observed. The expression of ABCG8, in particular, was markedly decreased. These findings were confirmed by RT-PCR analysis (Table 4). This is in contrast to the study in mice where ABCG5 expression was reported to be upregulated about 2-fold in response to dietary cholesterol [10]. In agreement with previous Northern blotting analysis, cholesterol feeding did not affect hepatic LDL receptor mRNA levels [18]. However, the rate of cycling and hence activity of the receptor is markedly decreased in response to dietary cholesterol [18]. 

Western blotting analysis showed that dietary cholesterol increases hepatic cholesterol 7*α* hydroxylase protein levels in rats but not in mice (Figure 1). Also, mice exhibited lower levels of cholesterol 7*α* hydroxylase protein in their livers. This is in agreement with a previous study [34] showing that dietary cholesterol caused a 3-fold increase in hepatic cholesterol 7*α* hydroxylase mRNA in rats with much less effect in C57BL/6J mice. Other reports showed that dietary cholesterol may act to increase or decrease cholesterol 7*α* hydroxylase mRNA levels in C57BL/6J mice depending on the type of fat added to the diet [35, 36]. Diets supplemented with olive oil (monounsaturated fatty acids) caused increased expression of hepatic cholesterol 7*α* hydroxylase. Western blotting analysis of cholesterol 7*α* hydroxylase was not preformed in these previous studies. 

In a recent study comparing the responses of hamsters and rats to cholesterol-enriched diets, it was found that hepatic cholesterol 7*α* hydroxylase expression in hamsters was not increased in response to cholesterol feeding [37]. This is in contrast with the increase seen in rats. However, hamsters do not adapt well to cholesterol supplemented diets, as their serum cholesterol levels are increased about 2-fold in response to a 0.1% cholesterol diet [37]. Also, hamsters express much lower (only 2 percent of that of rats) basal levels of hepatic HMG-CoA reductase [2], which renders them less able to resist the effects of dietary cholesterol [13]. 

The effect of a high cholesterol diet on the expression of genes in livers of mice has been previously investigated by microarray analysis [10]. In that study, Sqle was found to be downregulated 3.4 and 2.8-fold in males and females, respectively. This correlates with the decrease (7.9-fold) observed for hepatic Sqle expression in the present study. Apparently, no changes in CYP51 or cholesterol 7*α* hydroxylase expression were observed in the previous study [10]. HMG-CoA reductase was decreased 2-fold in males with no effect seen in females [10]. The greatest change in the previous study was observed for a disintegrin and metalloprotease domain 11 family member (Adam 11) which was upregulated 5 to 11-fold in the microarray and TaqMan analysis. ADAM 11 is a specific aggrecanase induced by interleukin 6 [38] which would promote an inflammatory response. In the present analysis, no change in Adam 11 expression was observed. A significant increase in serum amyloid A3, an acute phase reactant, was also observed in the earlier study [10]. An increase in this protein also indicates an inflammatory response to a high cholesterol diet.

The largest cholesterol-induced increase in hepatic gene expression in the present study was observed for insulin-like growth factor-binding protein 1 (IGFBP1), which was measured at over 4-fold by both microarray and RT-PCR analysis. This protein is produced by the liver during inflammation [39], possibly caused by dietary cholesterol. Increased levels are associated with an elevated risk of cardiovascular disease in type 2 diabetic patients [40]. IGFBP1 may also be a marker for insulin resistance [41] and be involved in the development of type 2 diabetes [42]. It may be protective against the growth of certain cancers by binding insulin-like growth factor-1, a potent mitogen [43]. 

In summary, a comprehensive examination of the adaptive hepatic transcriptional responses to dietary cholesterol was conducted using microarray and RT-PCR analysis. The data suggest that rats adapt, in part, to excess dietary cholesterol by markedly reducing hepatic cholesterol biosynthesis and enhancing elimination of cholesterol as bile acids due to induction of cholesterol 7*α* hydroxylase. A large increase in insulin-like growth factor-binding protein 1 was also observed. This could partially explain the inflammatory response associated with excess dietary cholesterol.

## Figures and Tables

**Table 1:** Oligonucleotide sequences used for RT-PCR analysis.

Gene	Primers	Sequence (5′→3′)
SQLE	Sense	AGTGAACAAACGAGGCGTCCTACT
	Antisense	AAAGCGACTGTCATTCCTCCACCA
CYP51	Sense	TTAGGTGACAACCTGACACACGCT
	Antisense	TGCTTACTGTCTTGCTCCTGGTGT
ABCG8	Sense	GATGCTGGCTATCATAGGGAGC
	Antisense	TCTCTGCCTGTGATAACGTCGA
ABCG5	Sense	TGAGCTCTTCCACCACTTCGACAA
	Antisense	TGTCCACCGATGTCAAGTCCATGT
ACAT2	Sense	TTGTGCCAGTGCACGTGTCTTCTA
	Antisense	GCTTCAGCTTGCTCATGGCTTCAA
CYP7A	Sense	TGAAAGCGGGAAAGCAAAGACCAC
	Antisense	TCTTGGACGGCAAAGAGTCTTCCA
7DHCR	Sense	TCAGCTTCCAGGTGCTGCTTTACT
	Antisense	ACAATCCCTGCTGGAGTTATGGCA
D14SR	Sense	AATGGTTTCCAGGCTCTGGTGCTA
	Antisense	ATAAAGCTGGTGAGAGTGGTCGCA
HMGCR	Sense	ATTGCACCGACAAGAAACCTGCTG
	Antisense	TTCTCTCACCACCTTGGCTGGAAT
HMGCS	Sense	TTGGTAGTTGCAGGAGACATCGCT
	Antisense	AGCATTTGGCCCAATTAGCAGAGC
IGFBP1	Sense	AGAGGAACAGCTGCTGGATAGCTT
	Antisense	AGGGCTCCTTCCATTTCTTGAGGT
LANS	Sense	ACTCTACGATGCTGTGGCTGTGTT
	Antisense	AAATACCCGCCACGCTTAGTCTCA
LIPIN2	Sense	TCTGCCATGGACTTGCCTGATGTA
	Antisense	ACTCGTGGTACGTGATGATGTGCT
PPAR*α*	Sense	AGACCTTGTGCATGGCTGAGAAGA
	Antisense	AATCGGACCTCTGCCTCCTTGTTT
PPAR*γ*	Sense	CAATGCCATCAGGTTTGGGCGAAT
	Antisense	ATACAAATGCTTTGCCAGGGCTCG
SC4MEOX	Sense	ACCTGGCACTATTTCCTGCACAGA
	Antisense	AGCCTGGAACTCGTGATGGACTTC

**Table 2:** Serum and liver cholesterol levels.

Condition	Serum	Liver
	Mg/100 mL	Mg/g
Normal	128 ± 5	3.2 ± 0.3
Cholesterol fed	102 ± 4	6.2 ± 0.5*

*P < 0.01 compared with normal chow fed rats. A 2-tailed distribution *t*-test, equal variance was used.

**Table 3:** Microarray analysis identifying genes upregulated by dietary cholesterol.

Normal			Cholesterol			Fold diff	Gene
386	507	439	2377	1264	2347	+4.5**	Insulin-like growth factor binding protein 1
476	457	536	2322	2418	988	+3.9*	B-cell leukemia/lymphoma 6 (predicted)
3597	5311	8493	16287	17914	12394	+2.7**	Cholesterol 7*α* hydroxylase
73	304	546	853	747	746	+2.5*	Similar to Ran-binding protein 2 (predicted)
2287	1733	1841	5590	5726	5166	+2.8**	Zinc finger protein 354A
127	387	320	756	494	773	+2.4*	PPAR*α*
253	372	250	766	759	536	+2.4**	Lipin 2 (predicted)
34	131	263	332	303	344	+2.3*	Zinc finger, matrin-like (predicted)
207	684	856	1358	1006	1277	+2.1*	HGPRTase
4255	2830	4209	9654	4959	8308	+2.0	Glucose-6-phosphatase

*P < 0.05; **P < 0.01. A 2-tailed distribution *t*-test, equal variance was used.

**Table 4:** Microarray analysis identifying genes downregulated by dietary cholesterol.

Normal			Cholesterol			Fold diff	Gene
1185	2381	1598	219	232	203	−7.9**	Squalene epoxidase
588	783	426	148	84	143	−4.8**	ABCG8
574	373	195	51	144	62	−4.5	Hypo protein XP_580018
2353	3066	2285	735	783	503	−3.8*	Acetyl CoA Acetyltransferase2
10377	11676	10752	2690	3350	3329	−3.5**	Lanosterol 14*α* demethylase
3890	4874	3668	929	1280	1426	−3.4**	Delta 14 sterol reductase
1049	893	1196	186	216	542	−3.3**	Cytokine inducible SH2 protein
238	316	242	65	115	76	−3.1**	Nuc factor, erythroid derived 2
733	1023	760	233	232	345	−3.1**	Ephrin A5
1998	608	524	340	343	375	−3.0	Dual specificity phosphatase1
11250	14322	9729	3511	4636	4315	−2.8**	Sterol C4 methyl oxidase-like
6083	6326	4997	1961	2597	2103	−2.6**	7-Dehydrocholesterol reductase
2851	2771	2112	1027	879	1055	−2.6**	Solute carrier family 25 mem 30
14079	17666	16412	5555	6296	7154	−2.5**	HMG-CoA Synthase 1
14890	13208	12147	5146	5920	5178	−2.5**	Farnesyl diphosphate synthase
1857	2636	1792	882	1025	635	−2.5**	Lanosterol synthase
4946	5502	4267	1503	2456	1667	−2.6**	Farnesyl diphos transferase 1
1695	2518	1567	870	834	814	−2.3*	ABCG5
13476	13702	14018	6048	7357	5541	−2.2**	Fatty acid desaturase 1
10599	8161	6620	3050	4215	4797	−2.1*	Hemato expressed homeobox
444	591	470	225	288	205	−2.1**	Acetoacetyl CoA synthase
1269	1465	1136	604	749	548	−2.0**	Hypo protein XP_579849
10087	11135	8727	4223	5304	5287	−2.0**	HMGCR
2516	2177	2744	1138	1049	1509	−2.0**	Forkhead box A2
848	1080	873	538	444	432	−2.0**	Mevalonate kinase

*P < 0.05; **P < 0.01. A 2-tailed distribution *t*-test, equal variance was used.

**Table 5:** RT-PCR analysis.

Gene	Normal	Cholesterol	Fold	*P* value
ABCG5	1.00 ± 0.07	0.67 ± 0.09	−1.49	0.018
ABCG8	1.02 ± 0.17	0.17 ± 0.03	−6.00	0.003
HMG-CoA synthase	1.03 ± 0.23	0.23 ± 0.01	−4.48	0.008
Squalene epoxidase	1.17 ± 0.26	0.14 ± 0.03	−8.29	0.002
Lanosterol 14*α* demeth	0.71 ± 0.28	0.16 ± 0.01	−4.53	0.027
Delta 14 sterol reductase	1.03 ± 0.31	0.19 ± 0.15	−5.33	0.013
Lanosterol synthase	1.12 ± 0.58	0.33 ± 0.14	−3.39	0.083
ACAT-2	1.00 ± 0.08	0.31 ± 0.07	−3.20	0.001
Sterol C4 Me Ox like	1.04 ± 0.31	0.39 ± 0.50	−2.65	0.128
7-Dehydrocholesterol red	1.01 ± 0.13	0.50 ± 0.06	−2.00	0.004
HMG-CoA reductase	1.02 ± 0.26	0.73 ± 0.28	−1.40	0.261
PPAR gamma	1.07 ± 0.40	1.02 ± 0.89	−1.04	0.944
PPAR alpha	1.01 ± 0.18	1.06 ± 0.44	+1.05	0.852
Lipin 2 (predicted)	1.03 ± 0.29	1.64 ± 0.42	+1.59	0.105
Chol 7*α* hydroxylase	1.17 ± 0.35	3.27 ± 0.97	+2.79	0.044
IGFBP1	1.02 ± 0.31	4.30 ± 1.90	+4.22	0.042

A 2-tailed distribution *t*-test, equal variance was used.

## Abbreviations

SQLE: Squalene epoxidase
CYP51: Lanosterol 14*α* demethylase
ABCG8: ATP binding cassette G8
ABCG5: ATP binding cassette G5
ACAT2: Acetyl CoA Acetyltransferase 2
CYP7A: Cholesterol 7*α* hydroxylase
7DHCR: 7-Dehydrocholesterol reductase
D14SR: Delta 14 sterol reductase
HMGCR: 3-Hydroxy-3-methylglutaryl coenzyme A reductase
HMGCS: 3-Hydroxy-3-methylglutaryl coenzyme A synthase
IGFBP1: Insulin-like growth factor binding protein 1
LANS: Lanosterol synthase
PPAR*α*: Peroxisome proliferator activated receptor *α*
PPAR*γ*: Peroxisome proliferator activated receptor *γ*
SCMEOX: Sterol C4 methyl oxidase-like.
